# CCNB2/SASP/Cathepsin B & PGE2 Axis Induce Cell Senescence Mediated Malignant Transformation

**DOI:** 10.7150/ijbs.63430

**Published:** 2021-08-13

**Authors:** Ying Wang, Hanbing Zhang, Minglei Wang, Jing He, Hui Guo, Lihua Li, Jie Wang

**Affiliations:** 1 Wuxi Cancer Institute, Affiliated Hospital of Jiangnan University, Wuxi 214062, Jiangsu, China.; 2 Department of Neurosurgery, Shanghai Deji Hospital, Qingdao University, Shanghai 200331, China.; 3 Department of Neurosurgery, PuTuo District People's Hospital, Shanghai 200060, China.; 4 Department of Pediatric Surgery, Guangzhou Institute of Pediatrics, Guangdong Provincial Key Laboratory of Research in Structural Birth Defect Disease, Guangzhou Women and Children's Medical Center, Guangzhou Medical University, Guangzhou 510623, Guangdong, China.

**Keywords:** CCNB2, cell senescence, Senescence-associated secretory phenotype, malignant transformation, glioma

## Abstract

Glioma is the most frequent and aggressive adult brain tumor with maximum mortality. However, the gene alteration and mechanism underlying malignant transformation of glioma remain largely unknown. We aimed to find key factors regulating tumor progression and malignant transformation of glioma. Here we compared the gene expression profiles of 693 glioma patients by HGG vs. LGG model, and identified a key factor CCNB2 for malignant transformation in glioma. CCNB2 induced a senescence-associated secretory phenotype (SASP) of glioma cells, and the malignant progression, such as invasion and excessive proliferation was mediated by secreting SASP cytokines, Cathepsin B and PGE2. These findings demonstrated a previously undiscovered link between senescence, CCNB2/SASP/Cathepsin B & PGE2 axis and malignant transformation in glioma. This might provide novel insights on developing new therapeutic regimens for abrogating aggressiveness of glioma.

## Introduction

Glioma is a most prevalent primary tumor of central nervous in adults 1, 2. According to the malignancy grade WHO I-IV and histological subtypes, glioma was categorized to Low-grade glioma (LGG) and High-grade glioma (HGG). HGG is highly malignant, easily recurrent, and the most lethal and difficult to treat, especially for glioblastoma (GBM). The 5 year overall survival of malignant glioma is no greater than 35% 2. High malignancy deteriorates the prognosis of patients and enhances the financial burden of society. How to find the key in malignant progression and restrain the aggressiveness of HGG is very important. Therefore, researches regarding the credible therapeutic targets and precise molecular mechanisms of malignant transformation in glioma have drawn extensive attention.

To solve this problem, we compared the gene expression profiles of 693 glioma cases by HGG vs. LGG model and chose 45 core genes in Top 1 module. In PPI network, a crucial core factor-CCNB2 and related pathway-cellular senescence were more attractive considering its high MCODE_score and intensive interactions in signaling pathway. Cellular senescence is a general phenomenon to prevent limitless cell proliferation. Its notable feature is the sustaining metabolic activity in growth retardation 3. Irreversible cell cycle arrest, cell swelling, Senescence-associated β-Galactosidase (SA-β-Gal) activation and less response to mitogen are hallmarks of senescence, which can be triggered by environmental stimuli 4, 5. Thus, induction of senescence is a potent protection against tumorigenesis and tumor progression originally. However, the defense mechanism of cellular senescence is a double-edged sword. Aged fibroblasts with senescence-associated secretory phenotype (SASP) are capable of remodeling a microenvironment for tumor development via secreting various cytokines 6, 7. Moreover, senescent tumor cells also present the pro-tumor potential by paracrine effects on surrounding non-senescent cells 8. Therefore, we further studied the role of CCNB2 on secreting of SASP cytokines and cell senescence mediated tumor aggressiveness. CCNB2, Cyclin B2, is a crucial component in cell cycle checking. It was reported promoting tumor malignancy such as radio-resistent in HGG (glioblastoma) 9, which was somewhat consistent with our findings.

**Figure 1:**
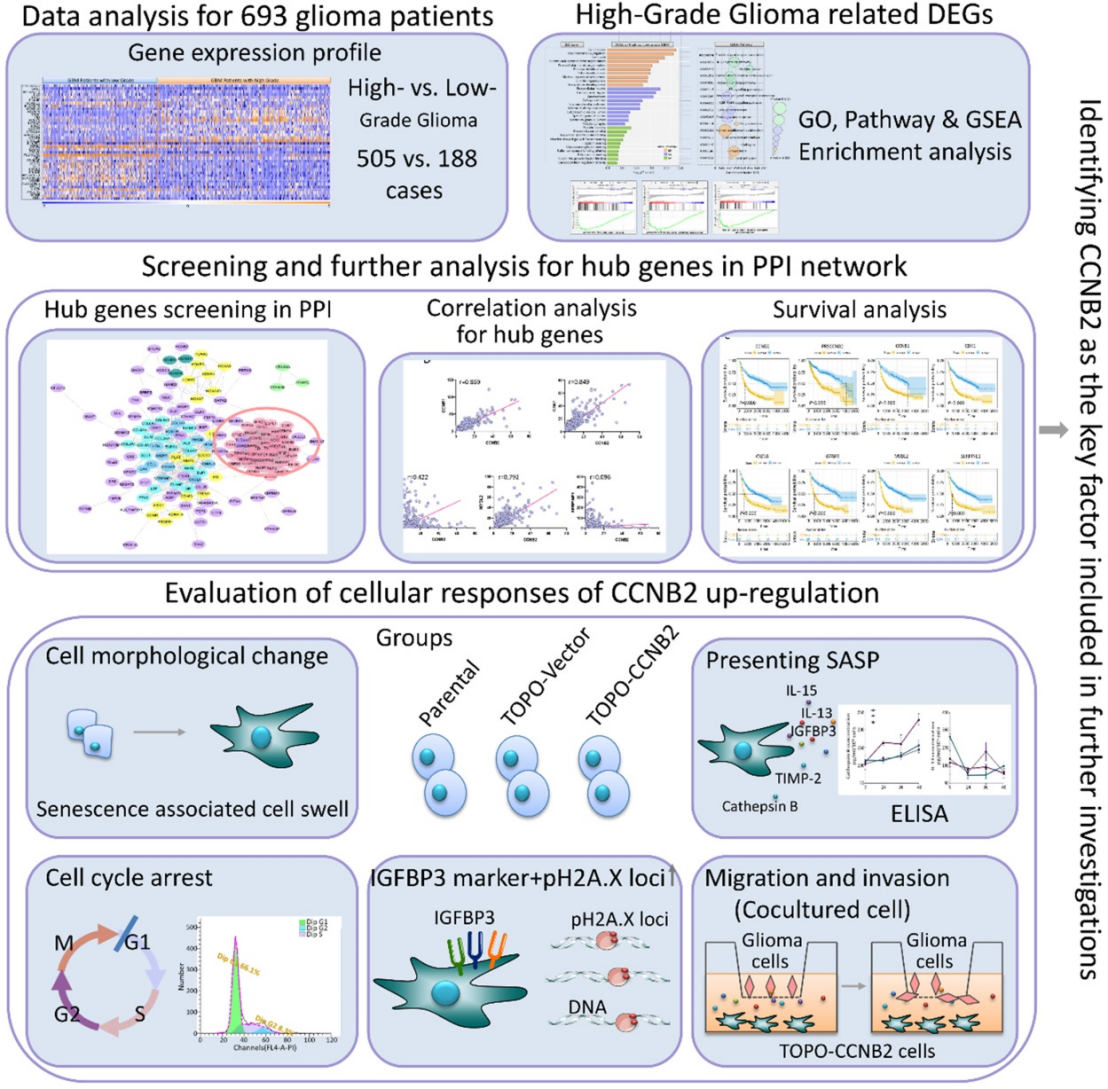
Flow diagram of the current study.

To the best of our knowledge, this study is the first one to screen key factors of malignant transformation in glioma, and proved crucial modulating roles of CCNB2/SASP/Cathepsin B & PGE2 axis on cell senescence mediated tumor aggressiveness (Figure 1). Revealing the complicated mechanisms within malignant progression of glioma will provide novel ideas on development of individualized therapeutic regimens.

## Results

### 510 differential expression genes (DEGs) were screened by HGG vs. LGG model

**Figure 2:**
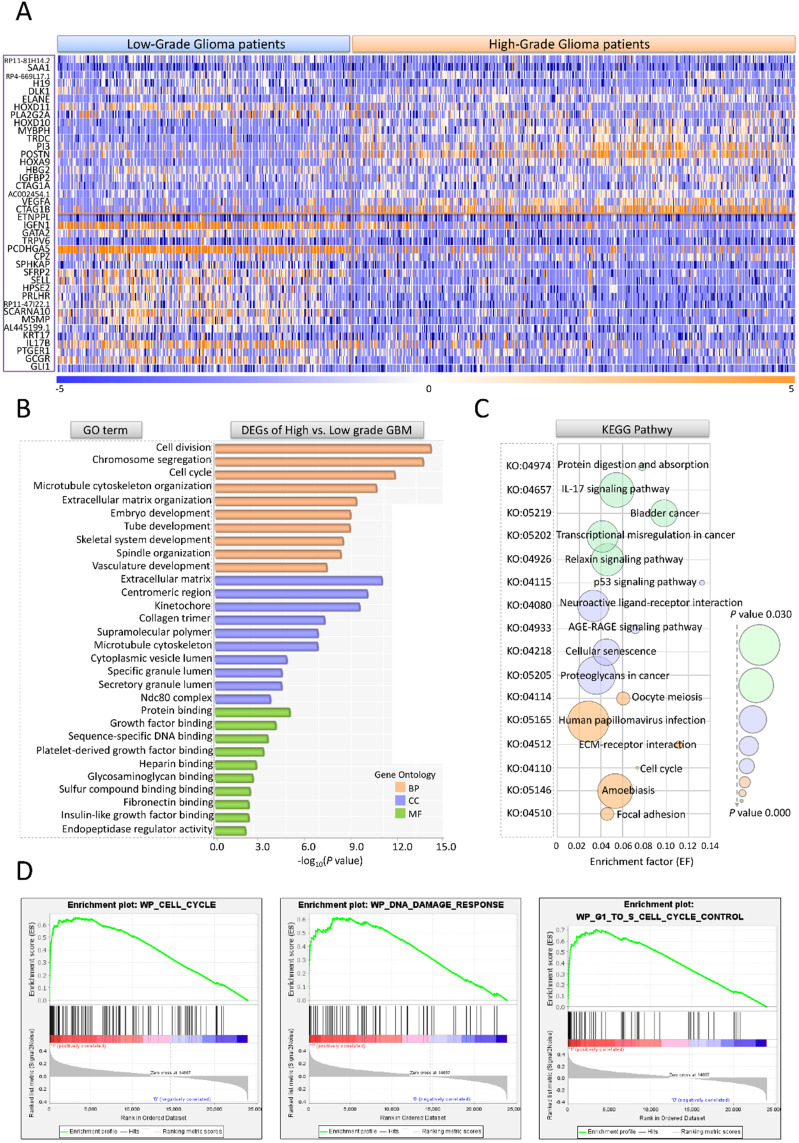
** DEG, GO, KEGG and GSEA enrichment analyses for III, IV stage glioma patients.** (A) The hierarchical cluster heatmap of DEGs differentiates HGG from LGG patients (HGG: n=505, LGG: n=188). Up and down-regulated genes are indicated with orange and blue, respectively. (B) GO analysis of DEGs. The y axis is the GO category. The x axis is -log_10_(*P* value). (C) KEGG pathway analysis of DEGs. The y axis refers to pathway term. The x axis is enrichment factor. (D) The major pathways in GSEA enrichment analyses.'

Gene expression profile, clinical characteristics and demographics of 693 glioma patients were screened from The Cancer Genome Atlas (TCGA) (https://www.cancer.gov/about-nci/organization/ccg/research/structural-genomics/tcga); (https://xenabrowser.net/datapages/) and The Chinese Glioma Genome Atlas (CGGA) (http://www.cgga.org.cn/) database. Among the 693 cases, 295 (42.6%) were female, 398 (57.4%) were male. Clinic-pathological analysis showed that 271 (39.1%) cases were astrocytic glioma, 249 (35.9%) were glioblastoma, 60 (8.7%) were oligodendroglioma, 112 (16.2%) were mixed glioma and 1(0.1%) was unknown type. For WHO tumor grade, 188 (27.1%) cases were WHO II (LGG, n=188), 256 (37.0%) were WHO III and 249 (35.9%) were WHO IV (HGG, n=505) 10. To assess the differences between HGG and LGG patients, the gene expression profiles of 693 glioma cases were compared using HGG vs. LGG model. There were 510 DEGs (FC>2, *P*<0.05) chosen out. The expression data for top 20 up/down-regulated genes in HGG and LGG groups were shown by hierarchical cluster heatmap (Figure 2A). Among all the DEGs, 470 genes containing DLK1, MYBPH, HOXD11, IGFBP2, VEGFA exhibited significantly up-regulation and 40 genes such as GLI1, GCGR, PTGER1showed notably down-regulation. We further performed GO and pathway analyses to illustrate the underlying function of HGG-related DEGs. The enrichment factor (EF) indicated the significance of particular function. In GO analysis, the DEGs were markedly enriched in 3 categories: (1) Biological process (BP): Cell division (GO: 0051301), Chromosome segregation (GO: 0007059), Cell cycle (GO: 0007049); (2) Cellular component (CC): Extracellular matrix (GO: 0031012), Centromeric region (GO: 0000779), Kinetochore (GO: 0000776); (C) Molecular function (MF): Protein binding (GO: 0005515), Growth factor binding (GO: 0019838), Sequence specific DNA binding (GO: 0043565). For KEGG pathway, HGG-related DEGs were evidently enriched in Protein digestion and absorption (KO: 04974), IL-17 signaling pathway (KO: 04657), AGE-RAGE signaling pathway (KO: 04933), Cellular senescence (KO: 04218) (Figure 2B, C). GSEA enrichment also suggested that cell cycle, DNA damage and G1 to S phase control are crucial in malignant transformation of glioma (Figure 2D).

**Figure 3:**
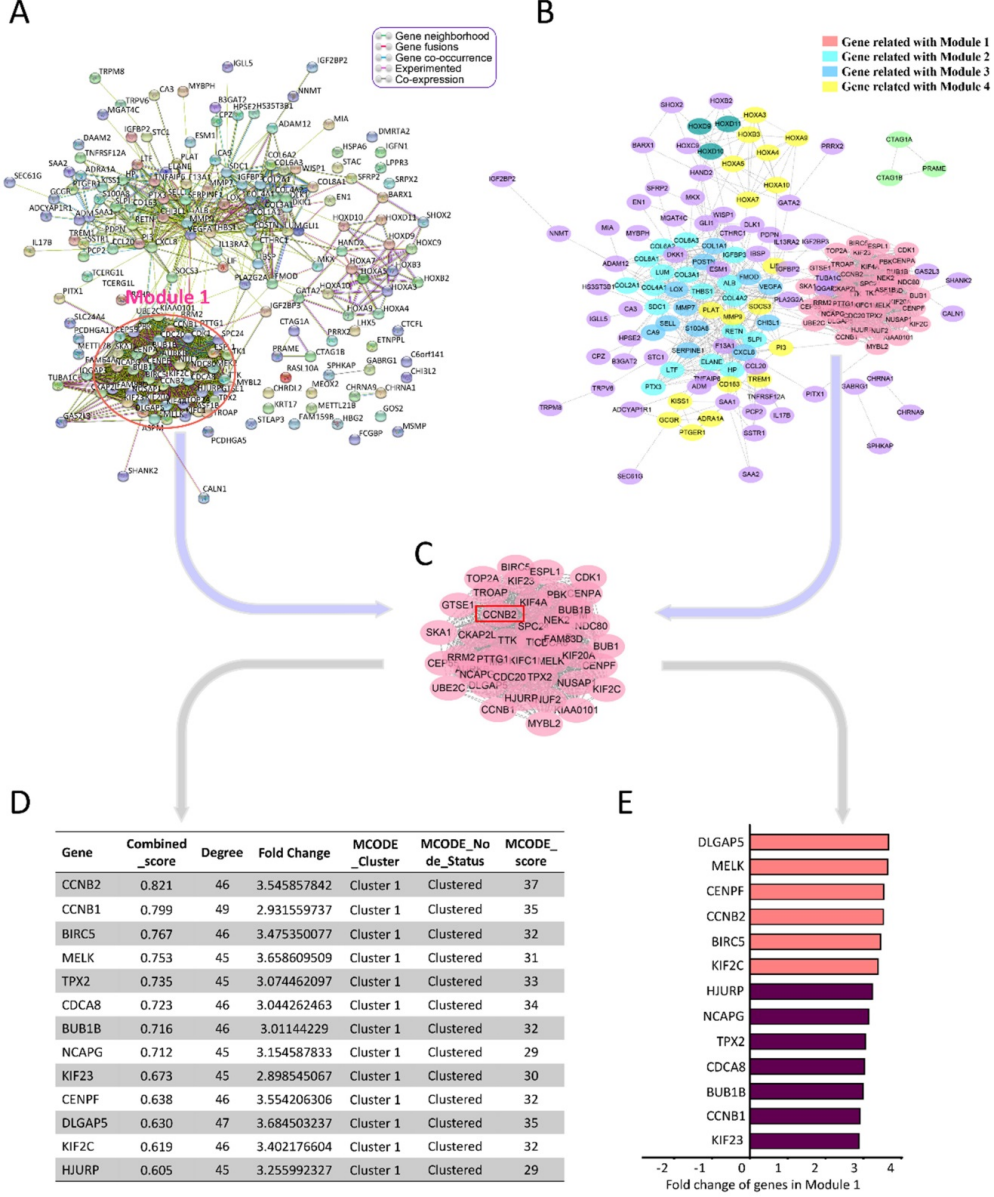
** PPI Network analyses of DEGs. (A)** PPI Network analysis by STRING online tools. Module 1 is circled with rose red. **(B)** PPI Network analysis applying Cytoscape 3.7.1 software. Module 1 includes genes with the highest M_CODE scores, which are indicated as pink round nodes. Module 2-4 are showed by cyan, blue, yellow nodes. **(C)** Genes in Module 1. **(D)** The characteristics of genes in Module 1. **(E)** The differentially expression FC (fold change) of genes in Module 1.

To better understand the advanced function of the DEGs, we conducted the protein-protein interaction (PPI) network analysis by STRING online tools (https://string-db.org/) and Cytoscape 3.8.0 (Figure 3A, B). HGG-related PPI network was composed of 4 major modules, containing 187 nodes and 1486 edges (PPI enrichment P<1.0e-16). Average node degree and average local clustering coefficient were 15.9 and 0.61, respectively. The top 1 module had 968 edges and 45 nodes, including CCNB2, CCNB1, BIRC5, MELK, TPX2 and CDCA8 (Figure 3C). The MCODE_scores of nodes in module 1 were the highest among all the modules (all score>25). They had the most intensive interactions in PPI network for HGG-related DEGs, suggesting the crucial cell-function-regulating roles of these nodes. Among them, combined_score (0.821) and MCODE_score (37) of CCNB2 were the biggest. Its FC was 3.55 (Figure 3D, E). Therefore, we chose CCNB2 for the further study. The potential impact of these malignant phenotype related genes for glioma deserved advanced investigations.

### Expression correlation and survival analyses for key gene CCNB2 in glioma malignant transformation

**Figure 4:**
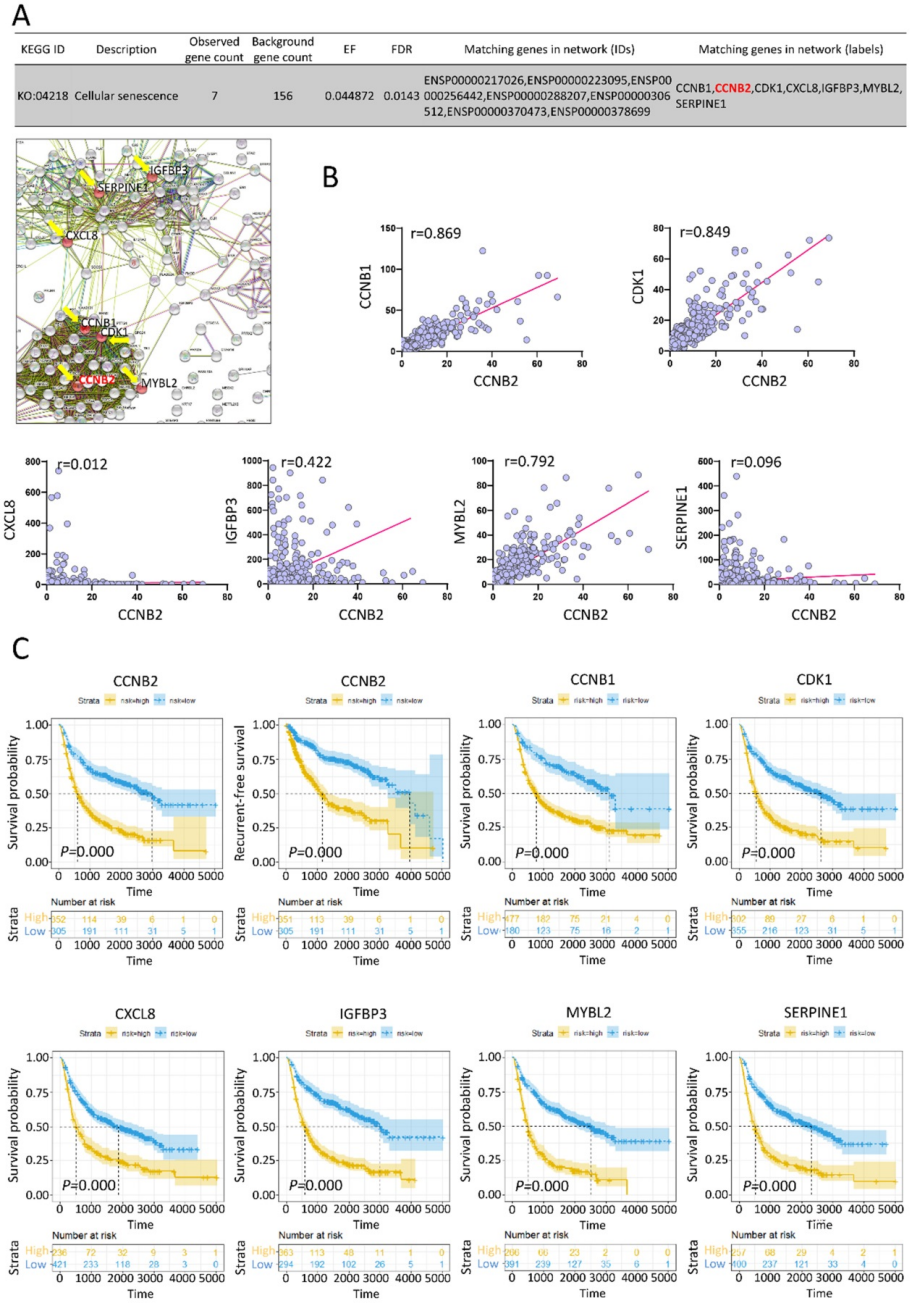
** Expression correlation and survival analyses for genes in CCNB2-associated cell senescence signal pathway. (A)** Matching genes in cell senescence pathway from PPI network. **(B)** Expression correlation analyses for genes in cell senescence pathway. **(C)** The Kaplan-Meier curve for the cell senescence genes in glioma patients. Glioma patients are categorized into high or low expression group according to gene expression folds. r: correlation coefficient.

To depict the potential function of CCNB2 in malignant transformation and malignant phenotype maintaining of glioma, we evaluated the expression correlation and prognostic value of CCNB2 and its related genes in PPI network. According to the KEGG pathway analysis, CCNB2 belonged to cellular senescence pathway (KO: 04218). Other PPI node genes in this pathway were CCNB1, CDK1, CXCL8, IGFBP3, MYBL2, SERPINE1 (as listed and indicated with arrows in PPI network, Figure 4A). Interestingly, IGFBP3 was just the cell marker for senescence. This finding suggested that CCNB2 was closely related with modulation of cell senescence. Expression correlation analyses also confirmed this point. CCNB1, CDK1, IGFBP3 and MYBL2 was significantly correlated with CCNB2 in expression level, with correlation coefficient 0.869, 0.849, 0.422 and 0.792 (r>0.3, *P*<0.05), respectively (Figure 4B).

Moreover, we evaluated the clinical predicting value of these genes. 693 Glioma patients with both gene expression and survival data were categorized into high or low expression group. Kaplan-Meier curves showed that high expression of CCNB2 significantly predicted poor prognosis for overall survival and recurrent-free survival (RFS) by log-rank test (HR>1, *P*=0.000<0.05) (Figure 4C). The upregulation of CCNB1, CDK1, CXCL8, IGFBP3, MYBL2, SERPINE1 also notably indicated poor overall survival of glioma patients (HR>1, *P*=0.000<0.05).

### CCNB2 facilitates G0/G1-phase arrest and cell swelling of glioma cells

Pathway analysis suggested that CCNB2 was a pivotal factor in cell senescence. Thus, we focused on this cellular biological process to perform deep explorations. Senescence is a general phenomenon to prevent limitless cell proliferation. Its notable feature is the sustaining metabolic activity in growth retardation. Cell cycle arrest, swelling and high-activity for SA-β-gal are important manifestations of senescent cells 3.

**Figure 5:**
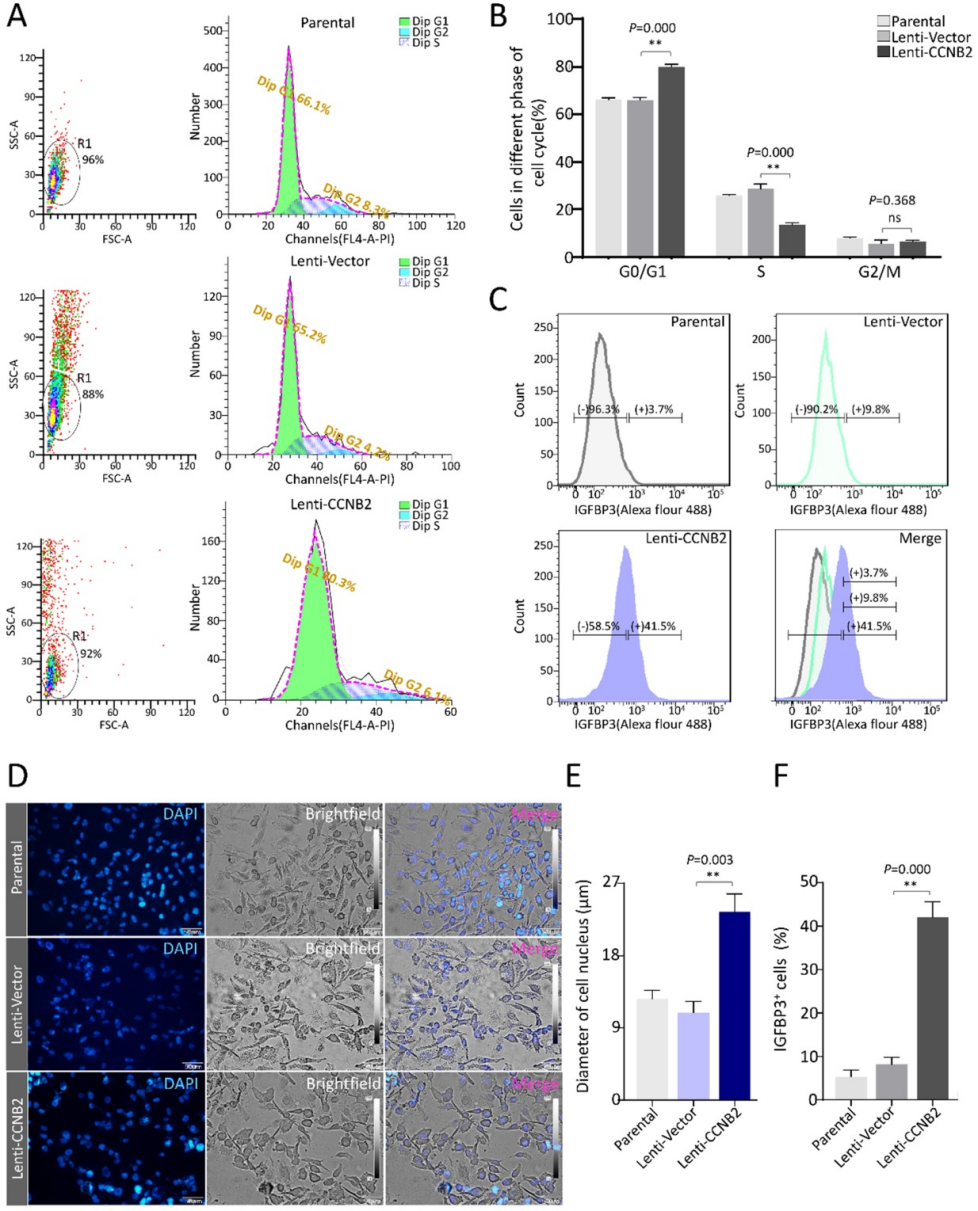
** CCNB2 facilitates G0/G1 phase arrest and cell swelling of glioma cells. (A)** Cell cycle distribution for glioma cells in Parental, Lenti-Vector and Lenti-CCNB2 groups. Left: SSC/FSC scatterplots for cell gating; Right: Histograms for cell cycle detection. **(B)** Quantitative analysis for cell percentages of G0/G1, S and G2/M phases from different groups. **(C)** The determination of cell marker for senescence-IGFBP3 using flow cytometry. **(D)** The morphological differences of glioma cells between different groups. Gray: light field, Blue: DAPI. **(E)** Quantitative comparison of average diameters of cell nucleus in different groups. **(F)** Quantification of IGFBP3^+^ cells from different groups. Scale bar: 50 µm. **:*P*<0.01; ns: not significant.

To confirm the role of key factor CCNB2 in senescence of glioma cells, we firstly detected the effect of CCNB2 on cell cycle and morphology. After ectopic expression of CCNB2 and cultivated for 7 days, glioma cells were stained with PI and examined on a flow cytometer. As shown in Figure 5A and 5B, CCNB2 induced an obvious G0/G1-phase arrest in contrast to Lenti-Vector group (79.9±1.19 vs. 66.1±1.08%, *P*=0.000), while the difference was not significant for G2/M-phase (6.5±0.51 vs. 5.6±1.45%, *P*=0.368). Furthermore, cell morphology was observed under an upright fluorescence microscope. Cells were significantly presenting swelling status in Lenti-CCNB2 group, with dilatated nucleus compared to Lenti-Vector group (23.7±2.28 vs. 11.8±2.05μm, *P*=0.003) (Figure 5D, E). These data suggested that CCNB2 notably facilitated G0/G1-phase arrest and cell dilatation, which contributed to cellular senescence. To assess whether senescence exactly occurred in the cultures, we examined the expression of IGFBP3, the typical cell marker for senescence. Consequently, IGFBP3^+^ cells were obviously increased in Lenti-CCNB2 group relative to control (41.9±3.56 vs. 8.1±1.66%, *P*=0.000) (Figure 5C, F). This finding confirmed the aging phenotype of cell cycle arrest and morphological changes.

### CCNB2 induces senescence and a SASP of glioma cells

**Figure 6:**
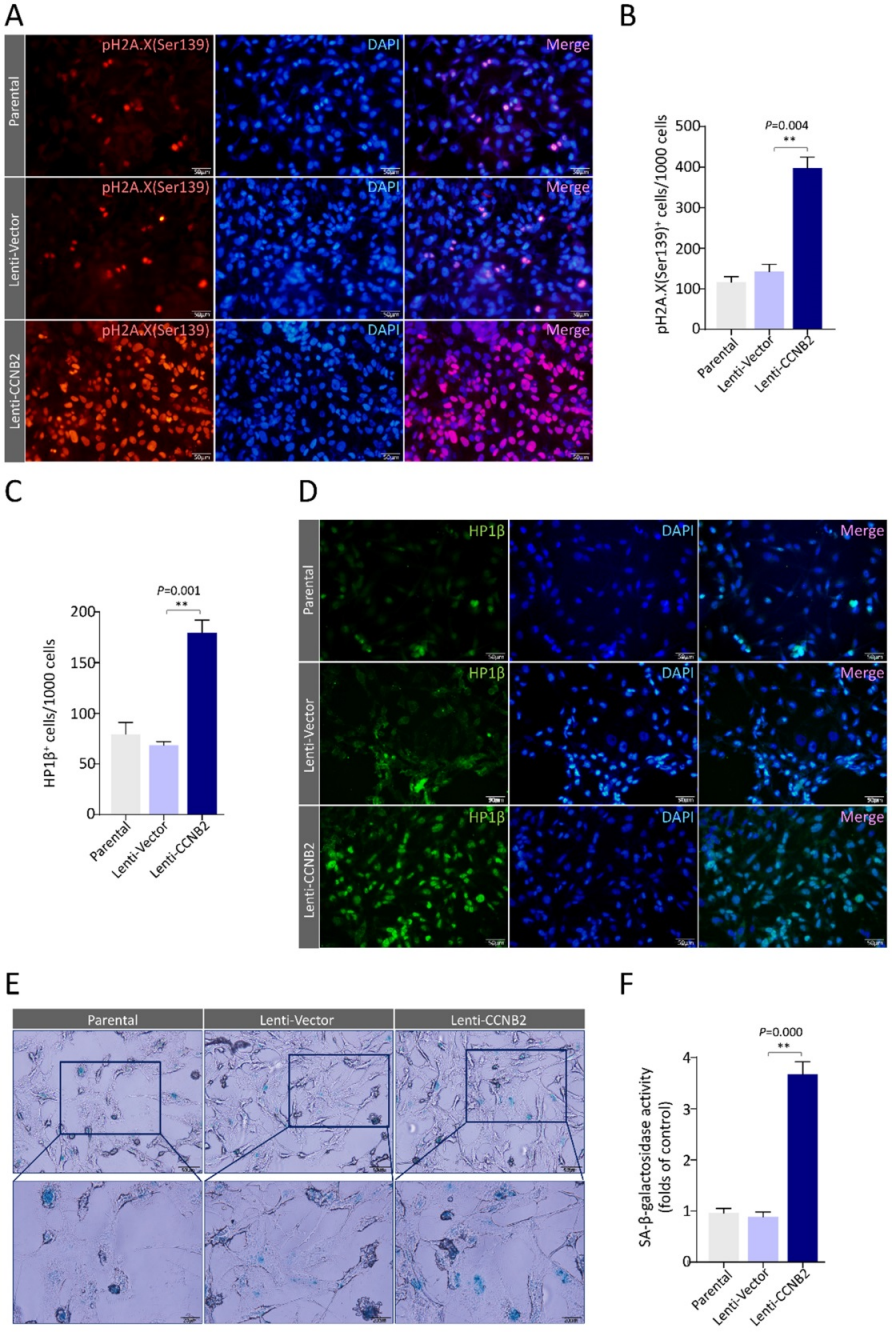
** CCNB2 induces senescence of glioma cells. (A)** Representative fluorescence‐microscopy photos of immunostaining for pH2A.X (Ser139) foci in Parental, Lenti-Vector, Lenti-CCNB2 groups and **(B)** corresponding quantitative analysis. Red: pγH2A.X (Ser139); Blue: DAPI. Scale bar: 50 µm. **(C)** Quantification of HP1β^+^ cells in different groups. **(D)** Fluorescence‐microscopy graphs of immunostaining for HP1β foci in Parental, Lenti-Vector, Lenti-CCNB2 groups. Green: HP1β; Blue: DAPI. Scale bar: 50 µm. **(E)** SA-β-gal staining for glioma cells in different groups. **(F)** Quantitative comparison of SA-β-Gal^+^ cells between different groups. Up panel: Scale bar: 50 µm. Down panel: Scale bar: 20 µm. **:*P*<0.01; ns: not significant.

To firmly establish the senescence signatures of CCNB2-overexpressed glioma cells, we tested two chromatin markers and a metabolic marker for senescence. The chromatin markers are phosphorylated histone H2A.X (Ser139) and heterochromatin protein 1β (HP1β) 11, 12. The accumulation of pH2A.X (Ser139) reveals high frequency of DNA double strand breaks (DSBs), an irreversible DNA damage 13. HP1β is a crucial component of heterochromatin foci. Using Immunostaining, we found that the pH2A.X (Ser139) foci were dramatically increased in CCNB2 ectopically expressed cells compared to control (369±57 vs. 152±24cells/1000 cells, *P*=0.004), indicating these cells becoming senescent (Figure 6A, B). Simultaneously, a similar phenomenon was observed in HP1β heterochromatin foci test (Lenti-CCNB2 vs. Lenti-Vector: 172±18 vs. 74±11 cells/1000 cells, *P*=0.001) (Figure 6C, D). We also examined the activity of the metabolic marker SA-β-Gal for senescence in different glioma cells. Cytochemical reaction results revealed that CCNB2 overexpression significantly led to an increase of SA-β-Gal activity (Lenti-CCNB2 vs Lenti-Vector: 3.7±0.25 vs. 0.8±0.18folds, *P*=0.000) (Figure 6E, F). According to the above, high level of CCNB2 definitely induced senescence of glioma cells. These findings were in consistent with the bioinformatics analyses for glioma TCGA data. However, we were facing with a crucial question: how did CCNB2-related senescent cells work in malignant transformation for glioma?

**Figure 7:**
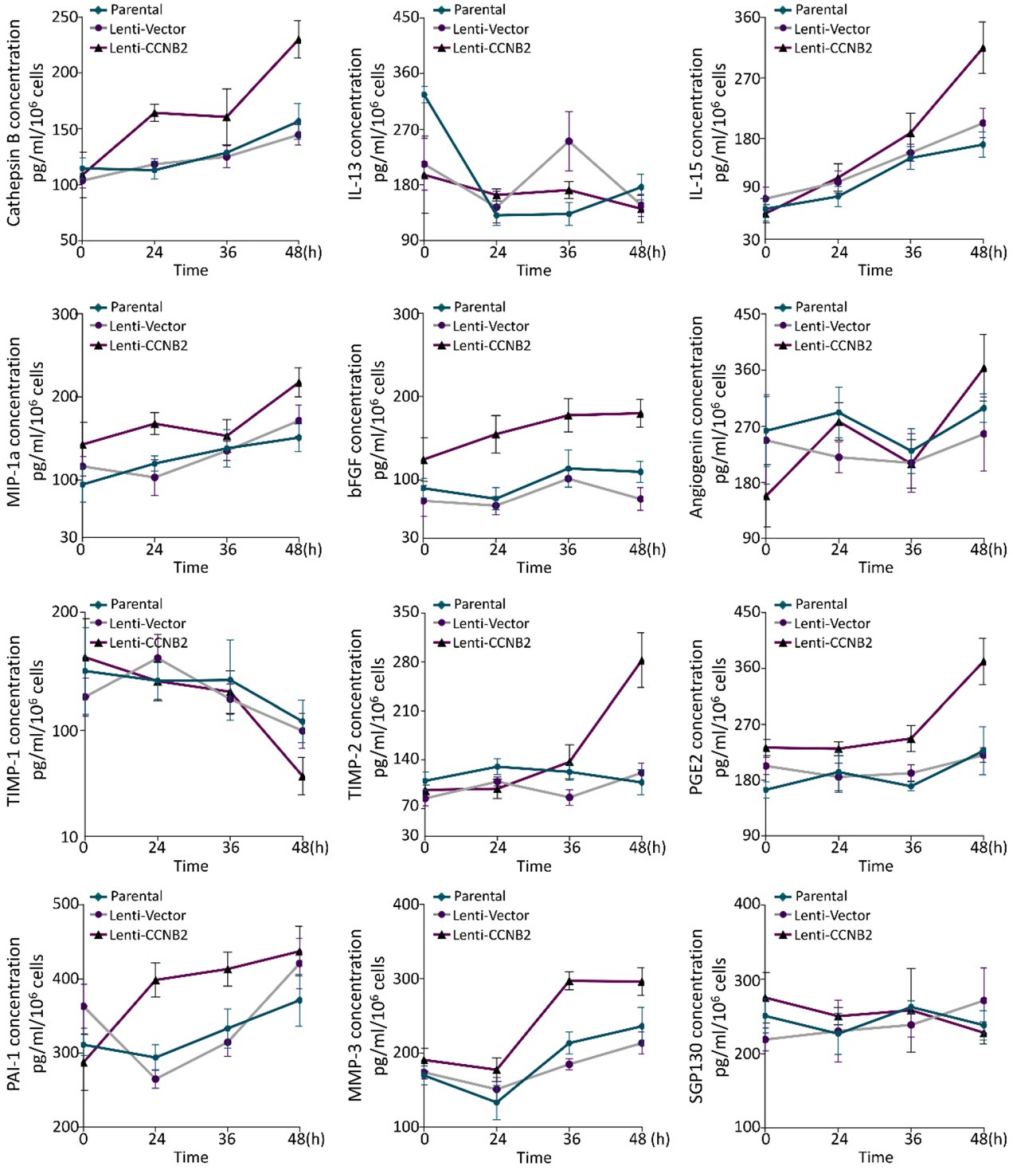
** The detection of senescent secretome markers in medium from glioma cells.** For instance, Cathepsin B concentration is detected using ELISA, in medium conditioned by glioma cells transducted with Lenti-Vector, Lenti-CCNB2 or not. Error bars, mean ± SD from three independent experiments.

Recently, increasing evidences showed that senescent tumor cells were capable of stimulating tumorigenesis and tumor progression via secreting various cytokines, which was called SASP. These cytokines would present paracrine effects on surrounding tumor cells 8, 14. To assess whether CCNB2 induced a SASP to promote the malignant transformation of glioma cells, we compared the cytokines concentration of medium from different groups by ELISA. These included cytokines are essential components of senescence secretome 8. As a result, Cathepsin B, bFGF, TMP2, PGE2 were significantly upregulated at 36 or 48h after senescence occurring (i.e., the 7^th^ day of CCNB2 overexpression was recorded as 0 hour in Figure 7). For instance, Cathepsin B at 48 h, the values of CCNB2 group in contrast to control was 230.0±16.70 vs. 144.7 ±9.29 pg/ml/10^6^ cells, *P*=0.002. These data corroborated that CCNB2 exactly induced a SASP of glioma cells, which might transmit messages for malignant transformation.

### CCNB2/SASP/Cathepsin B axis facilitates cell migration and invasion of glioma

**Figure 8:**
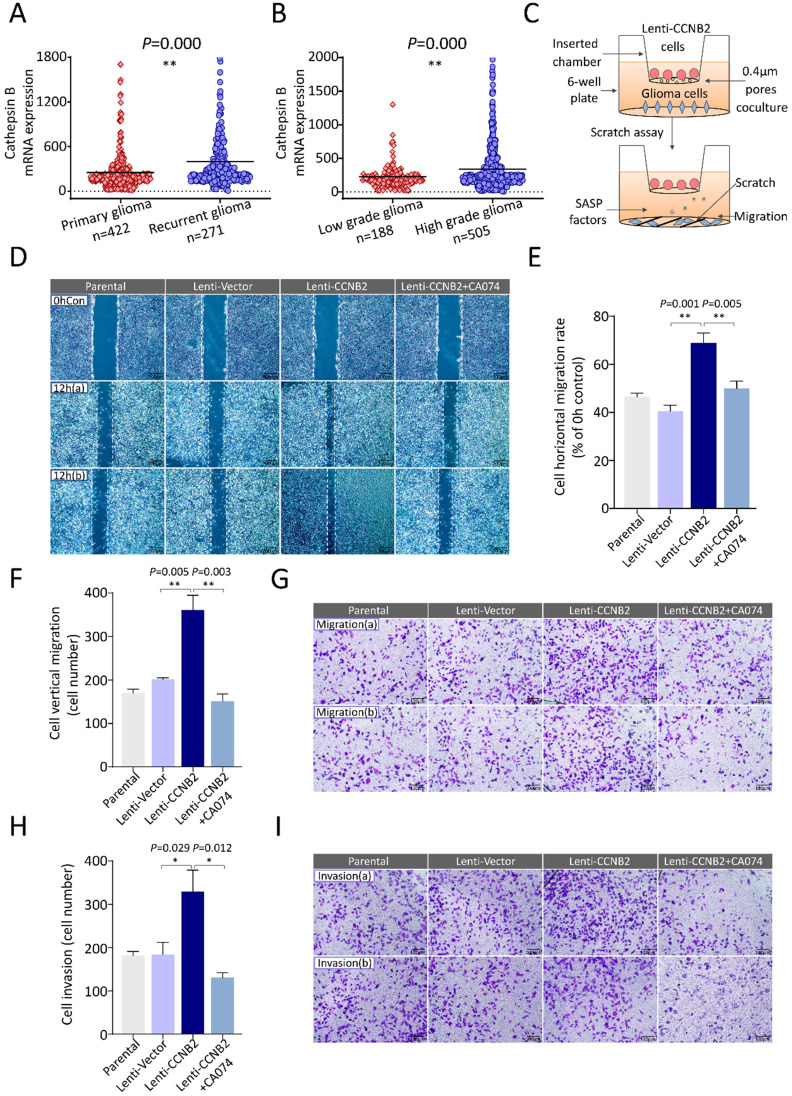
** CCNB2-mediated tumor cell metastasis could be abrogated by Cathepsin B inhibitor. (A)** Cathepsin B mRNA expression in patients with primary (n=422) or recurrent glioma (n=271). **(B)** Cathepsin B mRNA expression in patients with LGG (n=188) or HGG (n=505). **(C)** Schematic model of senescent cells coculture and migration induction system. **(D)** Representative graphs of scratch-wound assay in Parental, Lenti-Vector, Lenti-CCNB2, Lenti-CCNB2+CA074 (Cathepsin B inhibitor) groups and **(E)** corresponding quantitative analysis; (b) are duplicates. Scale bar: 200 µm. **(F)** Quantification of vertically migrated cells. **(G)** Representative images of migrated cells in transwell assay of different groups. Scale bar: 100 µm. **(H)** Quantitative comparison and **(I)** pictures of invaded cells in different groups. Scale bar: 100 µm. *:*P*<0.05, **:*P*<0.01.

To further explore the role of these SASP cytokines in glioma aggressiveness, we examined their effects on cell malignant transformation. Invasiveness and excessive proliferation underlie aggressiveness and are hallmarks of cancer malignancy. With screening, among these cytokines, Cathepsin B was considered as an invasion and metastasis promoter for tumor in generous studies 15-18. It was a lysosomal cysteine cathepsin. High level of Cathepsin B was correlated with dissociation from extracellular matrix and enhanced movement of tumor cells. Combined with our studies, Cathepsin B was significantly upregulated in recurrent glioma and HGG, compared to primary glioma and LGG, respectively (Figure 8A, B). These findings suggested that Cathepsin B might play pivotal roles in glioma metastasis, invasion and thus tumor malignant progression. Then we constructed a coculture cell model to measure the impacts of secreted Cathepsin B on migration and invasion of glioma cells (Figure 8C, Supplemental Figure 1). In scratch-wound healing assay, horizontal migration detection, coculture with Lenti-CCNB2 cells markedly facilitated the migration in contrast to vector group (69.5±3.91 vs. 42.3±3.57%, *P*=0.001). However, the migration potential was significantly inhibited by CA074, the inhibitor for Cathepsin B (Lenti-CCNB2+CA074 vs. Lenti-CCNB2: 47.9±5.57 vs. 69.5±3.91%, *P*=0.005), which suggested that Cathepsin B secreted from CCNB2-related senescent cells promoted tumor migration (Figure 8D, E). In transwell assay, the increase for vertical migration ability induced with SASP cytokines was remarkably abrogated by CA074 (Lenti-CCNB2+CA074 vs. Lenti-CCNB2: 160±22 vs. 344±44cells, *P*=0.003) (Figure 8F, G). Interestingly, the similar effect of CA074 was observed in invasion potential tests (Lenti-CCNB2+CA074 vs. Lenti-CCNB2: 143±25 vs. 308±61cells, *P*=0.012) (Figure 8H, I). Summarily, these senescent cells exhibited high metabolic activity and had the potential to influence the behavior of surrounding cells, which were nonsenescent.

### CCNB2/SASP/PGE2 axis promotes cell proliferation and 3D tumor spheroids formation of glioma

**Figure 9:**
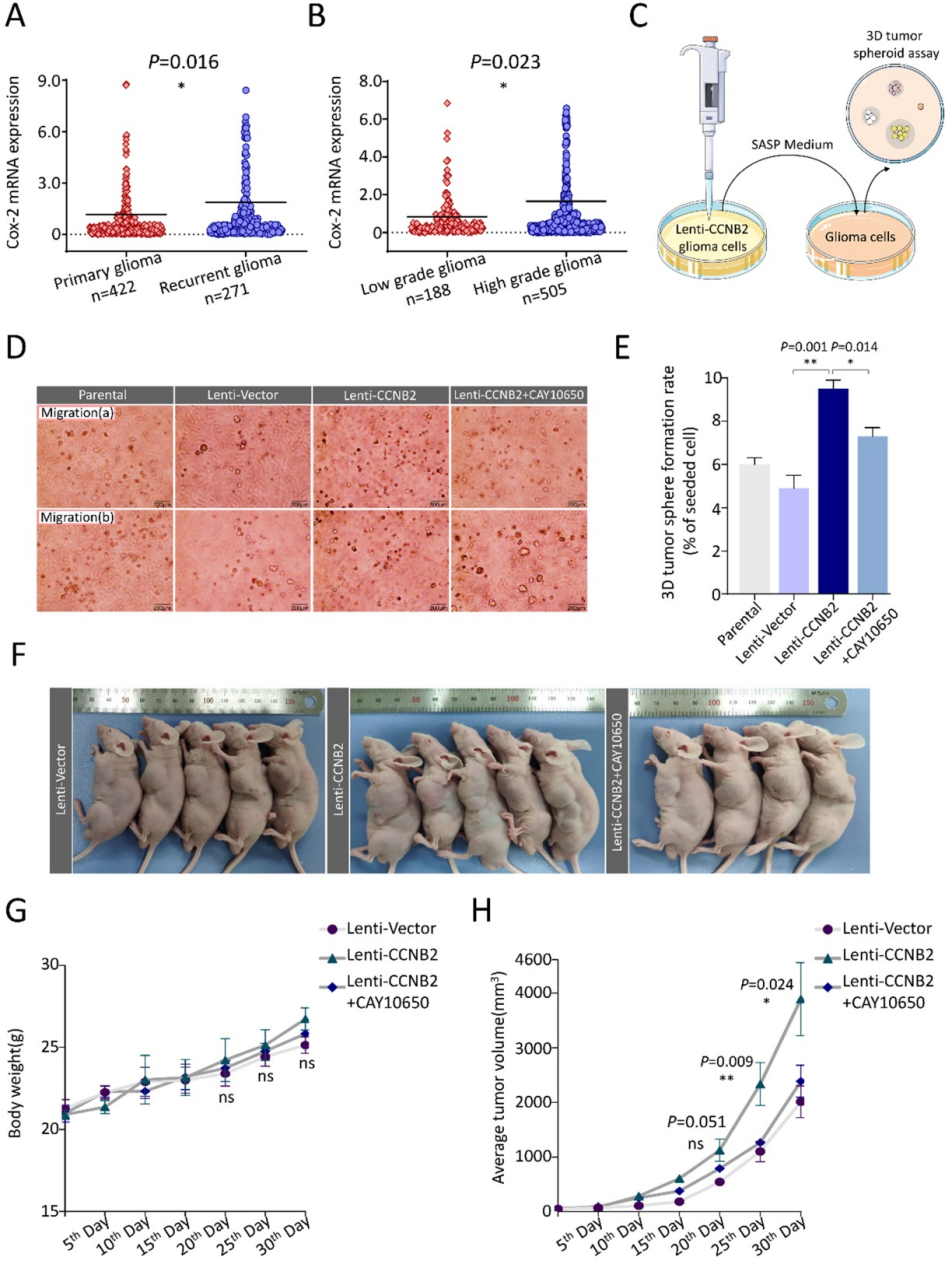
** CCNB2-mediated tumor cell proliferation could be abrogated by PGE2 inhibitor. (A)** mRNA expression of Cox-2 (PGE2 biosynthesis-related enzyme) in patients with primary (n=422) or recurrent glioma (n=271). **(B)** Cox-2 mRNA level in patients with LGG (n=188) or HGG (n=505). **(C)** Schematic model of 3D tumor spheroid culture system with SASP medium. **(D)** Representative images of 3D tumor spheroid assay in Parental, Lenti-Vector, Lenti-CCNB2, Lenti-CCNB2+CAY10650 (PGE2 inhibitor) groups and **(E)** corresponding quantitative analysis. Scale bar: 200 µm. **(F)** Representative graphs of nude mice xenograft tumors injected with Lenti-Vector medium, Lenti-CCNB2 medium or Lenti-CCNB2+CAY10650 medium. **(G)** Body weights for xenograft mice models of the three groups. **(H)** Average tumor volumes of the three groups. *:*P*<0.05; **:*P*<0.01; ns: not significant.

Another hallmark of malignancy is excessive proliferation. We also analyzed this influence of CCNB2-related senescent cells on other glioma cells. Within upregulated SASP cytokines of our ELISA assay, PGE2 (type 2 prostaglandin endoperoxide) was most correlated to tumor proliferation 19-21. More excitingly, the transcriptome data of PGE2 synthase- Cyclooxygenase-2 (Cox-2) also consolidated the crucial function of PGE2 in malignant transformation. Cox-2 expression was significantly higher in recurrent glioma and HGG than primary glioma and LGG, respectively (Figure 9A, B). We further examined the anchorage-independent proliferation of cells in conditioned medium using a 3D tumor spheroid culture model (Figure 9C). With treatment of medium from Lenti-Vector, Lenti-CCNB2 and Lenti-CCNB2+CAY10650 (inhibitor of PGE2 secretion) cells, SASP cytokines from Lenti CCNB2 supernatant significantly facilitate the 3D tumor sphere formation of glioma cells (Lenti-CCNB2 vs. Lenti-Vector: 9.3±0.53 vs. 4.6±0.79%, *P*=0.001), while it was remarkedly inhibited by CAY10650 (Lenti-CCNB2+CAY10650 vs. Lenti-CCNB2: 6.9±0.85 vs. 9.3±0.53%, *P*=0.014) (Figure 9D, E). The results meant that PGE2 was crucial in promoting anchorage-independent proliferation and thus malignant transformation of glioma cells, which worked through CCNB2/SASP/PGE2 axis in glioma cells.

Moreover, we constructed the animal models to investigate the effect of CCNB2/SASP/PGE2 axis on tumor growth *in vivo*. 24 Male nude mice were divided into 3 groups, bearing U251 xenografts intratumor injected with medium from Lenti-Vector, Lenti-CCNB2 or Lenti-CCNB2+CAY10650 cells. According to the growth kinetic curves, the propagation of tumors was dramatically accelerated by SASP cytokines in medium of Lenti-CCNB2 cells (30^th^ Day, Lenti-CCNB2 vs. Lenti-Vector: 3888.3±669.61 vs. 2012.7±289.99mm^3^, *P*=0.011). Nevertheless, this acceleration was obviously abrogated by PGE2 inhibition (30^th^ Day, Lenti-CCNB2+CAY10650 vs. Lenti-CCNB2: 2387.8±294.77 vs. 3888.3±669.61mm^3^, *P*=0.023) (Figure 9F, H). Body weight data of mice in different groups manifested that there was no significant change between them (Figure 9G). Our study authenticated the regulation role of CCNB2 on SASP cytokines secretion and tumor malignant transformation.

## Materials and methods

### Data extraction

The transcriptome profiles, demographics, clinical characteristics and survival Data of 693 glioma patients was searched and downloaded from The Cancer Genome Atlas Program (TCGA) (https://xenabrowser.net/datapages/) and Chinese Glioma Genome Atlas (CGGA) (http://www.cgga.org.cn/) databases. The RNA sequencing platform was Illumina HiSeq (Illumina, San Diego, CA, USA). Data was presented as RSEM values.

### DEG identification and GO, KEGG enrichment analysis

DEGs from HGG vs. LGG model was screened by R software (https://www.r-project.org/) and edgeR package (http://www.bioconductor.org/packages/release/bioc/html/edgeR.html). The filtration criteria of DEG were FC ≥ 2, *P*<0.05. GO and KEGG enrichment analyses were conducted using STRING online tools (https://string-db.org/). Protein-protein interaction (PPI) network was constructed by Cytoscape 3.8.0 software. In PPI analysis, Molecular COmplex DEtection (M_CODE) scores were calculated using topological clustering algorithm for detecting densely connected region of signaling regulatory modules.

### Cell culture and lentiviral vectors construction

U251 glioma cell line was purchased from American Tissue Culture Collection (ATCC) (Manassas, VA, USA) and cultured in DMEM plus 10% FBS (Gibco, NY, USA), 100U/ml penicillin and 100 μg/ml streptomycin (Beyotime, shanghai, China). To construct the lentiviral overexpression vector, a full-length of human CCNB2 cDNA clone was obtained from Genechem (Shanghai, China) and the CDS was inserted into pCDH-CMV-MCS-EF1-copGFP vector (CD511B-1, System Biosciences Inc., CA, USA) by EcoRI/BamH1 sites. Forward primer: 5'-GCGAATTCATGGCGCTGCTCCGA-3'; Reverse primer: 5'-GCGGATCCCTAGGACCTTCCTA-3'. Empty vector was used as control. Then, pCDH-CMV-MCS-EF1-copGFP-CCNB2 or empty vector, along with lentiviral package plasmids psPAX2 and pMD2.G (pCDH-CMV-MCS-EF1-copGFP-CCNB2 or empty vector: psPAX2: pMD2.G=8 μg: 6 μg: 4 μg), were transfected into 293T cells. Lentiviral particles in medium were purified and condensed by PEG8000. At last, pCDH-CMV-MCS-EF1-copGFP-CCNB2 or empty vector lentiviral transducted U251 cells were named as Lenti-CCNB2 or Lenti-Vector cells.

### Flow cytometry

Cell cycle detection: Day 1, cells were digested using 0.25% trypsin (Beyotime, shanghai, China), then fixed in 70% precooled ethanol, 4 °C overnight. Day 2, cells were washed by PBS and treated with PI (50 μg/ml) and Rnase A (0.1 mg/ml) (Beyotime, shanghai, China) in 37 °C, dark for 30 min. Subsequently, cells were run on BD FACSCanto^TM^ II flow cytometer (BD bioscience, CA, USA). Cell cycle distribution was analyzed by Flowjo 7.6.1 software (Tree Star Inc., Ashland, OR, USA).

Cell marker detection: Cells of Parental, Lenti-Vector or Lenti-CCNB2 group were digested (0.25% trypsin), washed (PBS) and fixed (70% precooled ethanol) for 2h. Following, they were washed and stained using Alexa Fluor® 488 labeled IGFBP3 antibody (sc-374365 AF488, 1:200, Santa Cruz Biotechnology, CA, USA) at 37 °C for 30min. Finally, the percentage of senescent cells was examined and analyzed with BD FACSCanto^TM^ II flow cytometer and Flowjo 7.6.1 software, respectively.

### Enzyme-linked immunosorbent assay (ELISA) of SASP cytokines

The concentration of SASP cytokines in medium conditioned by Parental, Lenti-Vector or Lenti-CCNB2 cells was measured applying Human Enzyme-linked Immunosorbent Assay Kits specific for the cytokine (R&D Systems, Minneapolis, MN, USA). All operations were down according to the manufacturer's instructions and the quantification of cytokines was performed on Synergy H1 Hybrid Multi-Mode Microplate Reader (BioTek Instruments, Winooski, VT, USA). The analysis software was Gen 5^TM^ 3.0 All-In-One Microplate Reader Software (BioTek Instruments, Winooski, VT, USA).

### Immunofluorescence analyses for pH2A.X and HP1β foci

Day1, cells were seeded on round cover slips in 6-well plates. Day 2, with 85% confluence, cells were fixed in 4% paraformaldehyde (10 min), permeabilized by 0.1% Triton X-100 (5 min) and blocked in 3% BSA (1 h). Then, they were incubated with Alexa Fluor® 594 anti-pH2A.X (Ser139) antibody (ab206898, 1:300, Abcam, Cambridge, MA, USA) or Alexa Fluor® 488 anti-HP1β antibody (sc-517288 AF488, 1:300, Santa Cruz Biotechnology, CA, USA MA, USA) overnight at 4 °C. Finally, cell nuclei were counterstained by DAPI (37 °C, 30 min). pH2A.X and HP1β foci were observed on Upright Fluorescence Microscope (Olympus BX53, Tokyo, Japan). Fluorescence quantification by average integral optical density were completed by Image J software (NIH, Bethesda, MA, USA).

### SA-β-Gal activity tests

The activity of SA-β-Gal in Lenti-CCNB2 cells was examined using SA-β-Gal staining kit (Beyotime, Shanghai, China) according to the manufacturer's recommendation. In brief, cells in 6-well plates were fixed, washed and treated by chromogenic substrate X-Gal overnight in 37 °C. After washing, the blue metabolic product of X-Gal, chloro-bromoindigo, indicating the activity of SA-β-Gal, was assessed using brightfield mode of Upright Fluorescence Microscope (Olympus BX53, Tokyo, Japan). Quantification was performed by calculating average integral optical density of the blue product with Image J (NIH, Bethesda, MA, USA).

### Transwell migration and invasion assay

Transwell migration and invasion assay was carried out using 24-well Transwell® units (8.0μm pore; Corning Inc., NY, USA). In brief, a coculture cell model for detection of migration and invasion potential was established. 5×10^3^ U251 cells in 150μl DMEM+10% FBS were added in upper inserts. To assess the influence of cytokines secreted by senescent or nonsenescent cells on neighboring glioma cells, 1×10^4^ Lenti-CCNB2 or Lenti-Vector cells in 500μl DMEM+10% FBS were filled in lower chambers. Different from migration inserts, invasion inserts were pre-coated with Corning Matrigel Basement Membrane Matrix (Corning Inc., NY, USA) (Supplemental Figure 1). After Incubation in 37℃ for 24 h, non-traversed cells in the inserts were wiped away. Migrated and invaded cells were fixed and stained in Crystal Violet Solution (Beyotime, Shanghai, China). Eventually, graphs were captured on an Inverted Fluorescence Microscope (Olympus IX71, Tokyo, Japan). Cells were counted using Image J (NIH, Bethesda, MA, USA). Each test was conducted in triplicate.

### 3D tumor spheroid assay

After 7 days of Lenti-Vector or Lenti-CCNB2 virus transduction (senescence occurring), the medium was collected and incubated with glioma cells in different groups. 3 days later, for lower layer, 100 μl pre-cooled Corning Matrigel Basement Membrane Matrix (5 mg/ml, Corning Inc., NY, USA) were added in 1well/24 well plate. To solidify the 3D culture Matrigel, the 24 well plates were maintained in 37 °C for 30 min. For upper layer, 1×10^4^ cells (100 μl) mixed with 200 μl pre-cooled Matrigel were filled in the well (37 °C, 30 min). At last, 500 μl conditioned medium of different groups was injected. After incubation for 10 days, 3D tumor spheroids were photographed by an Inverted Fluorescence Microscope (Olympus IX71, Tokyo, Japan) and calculated.

### Xenograft models construction

BALB/c nude mice (8 weeks old, male) was obtained from Shanghai Animal Center of Chinese Academy (Shanghai, China). Firstly, 1×10^7^ U251 cells were flank injected to 3 nude mice. When tumor size was ~100 mm^3^, the tumors was cut into 8mm^3^ globules and inoculated to the right flank of nude mice. Secondly, Animals were randomly categorized into 3 groups (n=8/group) while xenografts reached ~50 mm^3^. The medium conditioned by Lenti-Vector, Lenti-CCNB2 and Lenti-CCNB2+CAY10650 cells was intratumorally injected to mice in corresponding group every 2 days. Tumor volume and body weight of these mice were monitored every 5 days. The animal study protocol was approved by the ethics committee of Shanghai Deji Hospital.

### Statistical Analysis

Statistical analyses were two-sided and performed by GraphPad Prism 8.0 (GraphPad Software, La Jolla, CA, USA) or Statistical Program for Social Science (SPSS 18.0, Chicago, IL, USA). All data were written as mean ± SD. Variance between different groups was calculated using Student's t test (two groups) or one-way ANOVA (multi groups). Log-rank tests was applied to assess the differences of survival analyses. *P*<0.05 was identified as statistically significant in the current study.

## Discussion

In this study, we identified a key factor CCNB2 and CCNB2/SASP/Cathepsin B & PGE2 axis inducing cell senescence mediated malignant transformation in glioma.

Firstly, with TCGA and CGGA data mining, global gene expression profiles of 693 glioma cases were compared by HGG vs. LGG model. There were 510 malignant transformation-related DEGs extracted out. We further performed GO and pathway analyses to illustrate the underlying function of DEGs. GO analysis indicated Cell division, Cell cycle and so on. For KEGG pathway, malignant transformation-related DEGs were evidently enriched in protein digestion and absorption, AGE-RAGE signaling pathway, Cellular senescence etc. PPI network analysis generated 4 major modules. The top 1 module included CCNB2, CCNB1, BIRC5, MELK, TPX2 and CDCA8. Interestingly, these core genes are all participating in modulation of cell cycle, cell division or cell senescence. Simultaneously, they were also play roles in malignant progression of tumor 22-27. Among them, CCNB2 was chosen for the functional study due to its most intensive interactions in signaling pathway. The potential impact of this malignant phenotype related factor in glioma deserved advanced investigations.

Subsequently, we evaluated the expression correlation and prognostic value of CCNB2 and its related genes in cellular senescence pathway. CCNB1, CDK1, IGFBP3 and MYBL2 was significantly correlated with CCNB2 in expression level. Survival analysis indicated that high expression of CCNB2 significantly predicted poor prognosis for overall survival and recurrent-free survival (RFS) of glioma. That is to say, CCNB2 was a pivotal regulator of cell senescence, and implicated in malignant transformation.

Additionally, the phenotypes of senescence, such as G0/G1-phase arrest and cell swelling were authenticated in CCNB2 glioma cells. To assess whether senescence exactly occurred, we confirmed the expression of IGFBP3, the typical cell marker for senescence. The chromatin markers, pH2A.X (Ser139) and HP1β, and the metabolic marker SA-β-Gal were also identified in CCNB2-overexpression cells. These evidences were in consistent with the bioinformatics analyses for glioma patients.

Senescence is a double-edged sword. Originally, induction of senescence by environmental stimuli is a potent protection against tumorigenesis and tumor progression 11. However, aged cells are capable of remodeling a microenvironment for tumor development via secreting various cytokines 6, 7. ELISA data revealed that CCNB2 indeed induced a SASP including various cytokines such as growth factor and proteinases. Cathepsin B, bFGF, TMP2 and PGE2 were all significantly upregulated. To test whether these cytokines transmitted messages for malignant transformation, we examined their effects on cell invasiveness and excessive proliferation. Those were hallmarks of cancer malignancy. With clinical information and expression profiles analysis, we found that Cathepsin B and PGE2 synthase-Cox-2 was significantly upregulated in recurrent glioma and HGG, compared to primary glioma and LGG, respectively. These data indicated that Cathepsin B and PGE2 might play pivotal roles in glioma malignant progression. Scratch-wound healing and transwell assays demonstrated that secreted Cathepsin B from CCNB2-related senescent cells obviously facilitated migration and invasion of glioma cells. Using inhibitor, PGE2 in CCNB2 medium was verified to enhance proliferation potential of glioma *in vitro* and *in vivo*, which worked through CCNB2/SASP/PGE2 axis in glioma cells. Conclusively, these senescent cells exhibited high metabolic activity and had the potential to influence the behavior of surrounding cells, which were nonsenescent. Similarly, for epithelial tumor cells, researchers found that senescent fibroblasts could drive tumor progression, such as cell proliferation and vascularization through SASP 28. This phenomenon was called maladaptive senescence 28-31.

**Figure 10:**
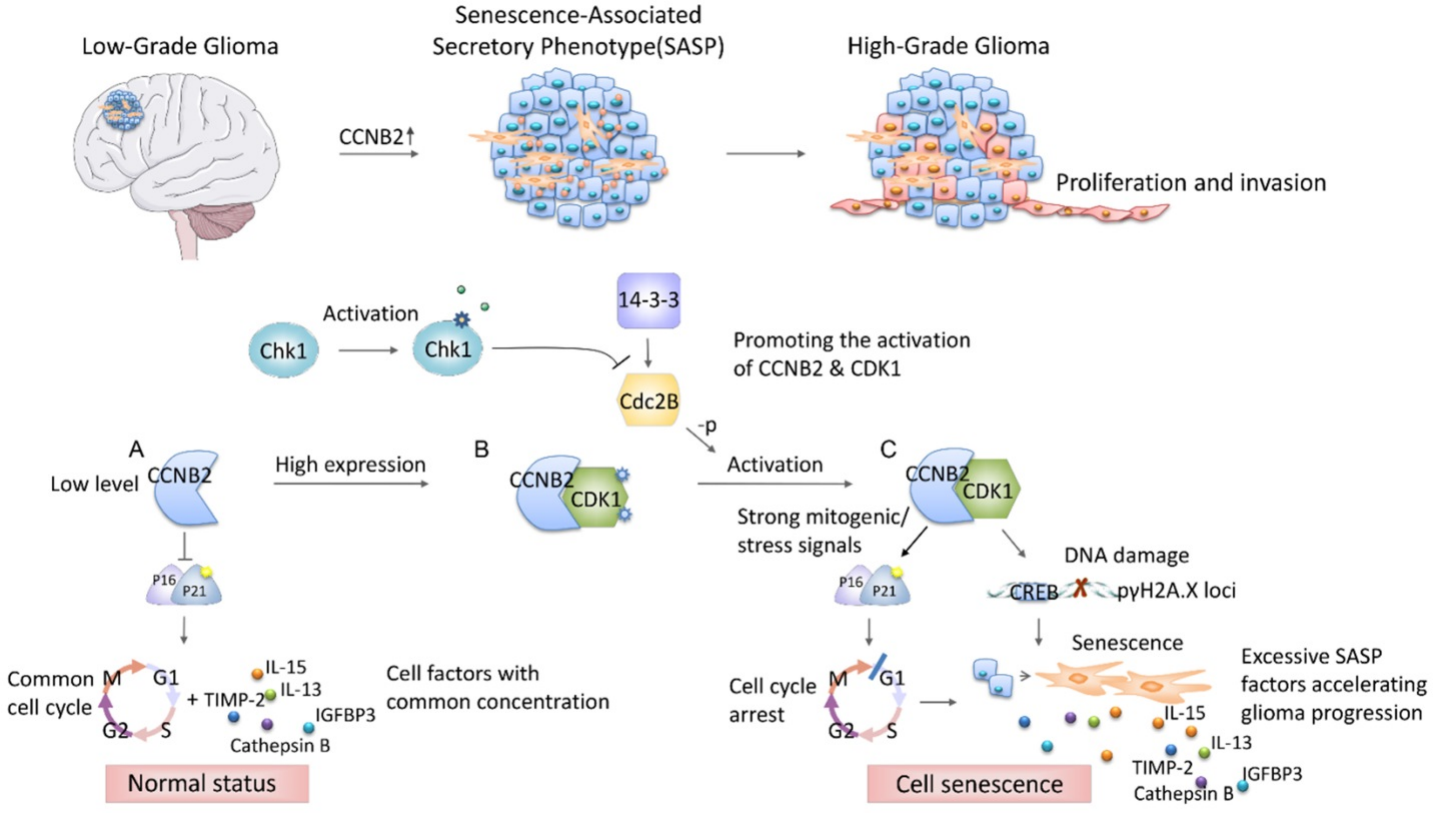
** Schematic diagram depicting the potential role of CCNB2 in modulating cell senescence and progression of glioma.** In the progression from low to high grade glioma, the malignant phenotype of tumor cells is induced by CCNB2 mediated SASP. CCNB2 might modulate cell senescence via initiating the mitogenic/ stress signals (A-C), thus facilitate the activating of p16/p21 complex, and γH2A.X phosphorylation. Subsequently, it further enhances relative SASP factors production and the deterioration of tumor microenvironment, which significantly accelerates cell malignant transformation and progression of glioma.

Summarily, this study demonstrated, for the first time, that CCNB2 functioned as a malignant transformation regulator and a tumor promoter of glioma. We extracted a series of malignant phenotype-related genes from gene expression profiles of 693 glioma patients. Then we identified the key factor-CCNB2 which significantly promoted invasion and excessive proliferation of glioma through CCNB2/SASP/Cathepsin B and CCNB2/SASP/PGE2 axis. In the development from Low to High-grade glioma, CCNB2 enhanced SASP cytokines production and the deterioration of tumor microenvironment, which significantly accelerated cell malignant transformation and progression of glioma (Figure 10).

Our findings might arouse novel insights on decoding the complicated mechanism of malignant transformation and developing new therapeutic regimens for glioma. This investigation would also be benefit for the prediction of prognosis, along with target therapy development for the malignant transformation of glioma. In conclusion, the correlation of CCNB2/SASP/Cathepsin B & PGE2 axis and senescence mediated malignant transformation in glioma is valuable enough to warrant advanced explorations.

## Supplementary Material

Supplementary figure S1.

Supplementary tables.

## Abbreviations

SASP: Senescence-associated secretory phenotype
SA-β-Gal: Senescence-associated β-Galactosidase
TCGA: The Cancer Genome Atlas
LGG: Low-grade glioma
HGG: High-grade glioma
DEG: Differential expression gene
FC: Fold change
GO: Gene ontology
KEGG: Kyoto Encyclopedia of Genes and Genomes
GSEA: Gene set enrichment analysis
PPI: Protein-protein interaction
EF: Enrichment factor
CCNB2: Cyclin B2
PGE2: Type 2 prostaglandin endoperoxide
