# Supplementary material for: CCNB2/SASP/Cathepsin B & PGE2 Axis Induce Cell Senescence Mediated Malignant Transformation
Source: Int J Biol Sci. 2021 Aug 13;17(13):3538–53. doi: 10.7150/ijbs.63430 (PMC8416730; doi:10.7150/ijbs.63430)
Supplement: Supplementary file 1 — Supplementary figure S1. [file ijbsv17p3538s1.pdf]

Supplemental figure 1:

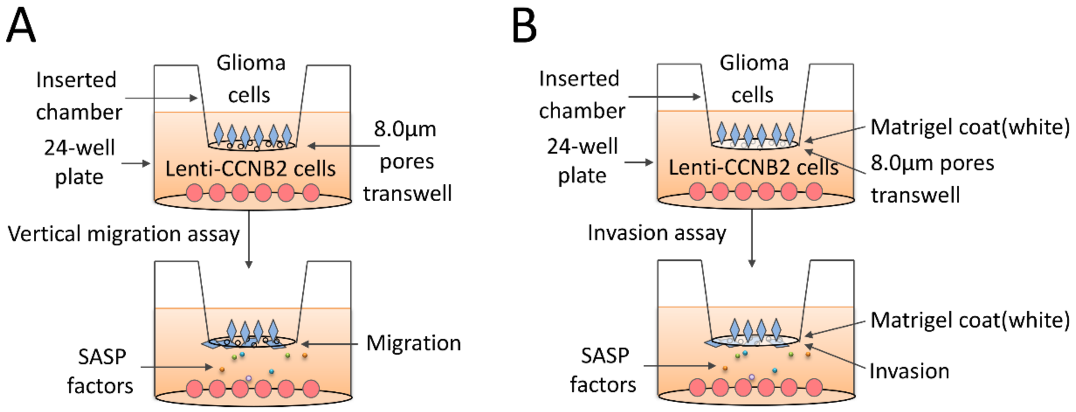


**Supplemental figure 1. Schematic model of vertical migration and invasion assay system.**
